# Sortie précoce en post-partum: résultats et facteurs de risque de ré hospitalization

**DOI:** 10.11604/pamj.2016.24.189.9371

**Published:** 2016-07-01

**Authors:** Mehdi Kehila, Khaoula Magdoud, Omar Touhami, Hassine Saber Abouda, Sara Jeridi, Sofiène Ben Marzouk, Sami Mahjoub, Rim Ben Hmid, Mohamed Badis Chanoufi

**Affiliations:** 1 Service C du Centre de Maternité et de Néonatologie de Tunis, Faculté de Médecine de Tunis, Université Tunis El Manar, Tunisie; 2 Service d’Anesthésie-Réanimation du Centre de Maternité et de Néonatologie de Tunis, Faculté de Médecine de Tunis, Université Tunis El Manar, Tunisie

**Keywords:** Post-partum, hospitalisation, accouchement, sortie précoce, Postpartum, hospitalization, childbirth, early discharge

## Abstract

Cette étude nous permettra d'évaluer la pratique d'une sortie précoce en post-partum en analysant le taux de réadmission maternelle et en essayant d'identifier les facteurs de risque de ré hospitalisation. Il s'agit d'une étude prospective et analytique à propos de 1206 patientes sorties de l'hôpital à J1 du post-partum. Pour chaque patiente, nous avons noté les données épidémiologiques, le déroulement de la grossesse et de l'accouchement. Nous avons identifié les causes des ré hospitalisations ainsi que leur évolution. Le taux de césariennes était de 42%. Le taux de réadmissions maternelles était de 0,99%. La durée moyenne du séjour lors de la ré hospitalisation était de 26 heures. Les troubles du transit ont été le motif de consultation le plus fréquent (50% des cas) suivis par la fièvre (25% des cas). Les facteurs de risque de ré hospitalisation, identifiés dans notre étude étaient: la césarienne (p=0,004), la césarienne en urgence (p=0,016), l'anémie (p<0,001) et la thrombopénie (p=0,003). La sortie précoce en post-partum semble une option sure pour la maman et le nouveau-né sous réserve d'une information claire de la patiente et du respect des critères de sélection.

## Abstract (en)

The objective of this study is to evaluate the practice of early postpartum discharge by analyzing maternal readmission rates and identifying readmission risk factors. This is a prospective and analytical study of 1206 patients discharged from hospital on postpartum day 1. For each patient we collected the epidemiological data, the course of pregnancy and childbirth. We identified the causes of readmission and their evolution. Cesarean delivery rate was 42%. Maternal readmission rate was 0.99%. The average length of stay in hospital after readmission was 26 hours. Intestinal transit disorders were the most frequent reason for consultation (50% of cases) followed by fever (25% of cases). The readmission risk factors identified in our study were: cesarean section (p = 0.004), emergency cesarean section (p = 0.016) anemia (P < 0.001) and thrombopenia (p = 0.003). Early postpartum discharge seems a safe option for the mother and their newborn children subject to the ability to clearly communicate health information to the patient and to the compliance with selection criteria.

## Introduction

Depuis les années 70 et plus tôt dans certains pays occidentaux, on a assisté à une diminution remarquable de la durée d'hospitalisation en post-partum. Dans certains pays comme les Etats-Unis, le Canada, l'Australie, le Royaume uni et la Suède, la durée d'hospitalisation est passée de quatorze jours dans les années 50 à une durée de 3 à 4 jours pour les accouchements par voie basse sans complication. Vers le milieu des années 90, la durée de l'hospitalisation dans certaines régions des états unis a atteint 12 à 24h pour les accouchements par voie basse et 48 à 72h pour les accouchements par césarienne [1, 2]. Il y a eu beaucoup de controverses au sujet de la courte hospitalisation et ses risques potentiels pour la mère et le nouveau-né. La question s'est posée depuis la fin des années 50 avec la publication par Hellman du premier essai randomisé évaluant cette pratique [3]. Les effets négatifs redoutés lors d'une hospitalisation de courte durée sont essentiellement un retard de diagnostic et de mise en route du traitement aussi bien pour les pathologies maternelles que néonatales, une incidence plus élevée des problèmes d'allaitement conduisant à un sevrage précoce, une augmentation du taux de dépression en post-partum et une augmentation du taux de ré hospitalisations maternelles et néonatales [1, 4, 5] La courte durée d'hospitalisation a été par contre encouragée dans certains centres de naissance car elle permet le passage d'un contexte de malade dans une maternité à une approche plus centrée sur la famille [5, 6]. Elle offre également la possibilité aux mères d'avoir plus de repos et de sommeil dans leur propre maison [4]. Une courte durée d'hospitalisation permet également de diminuer le temps d'exposition de la maman et du nouveau-né aux infections nosocomiales [3], elle augmente la confiance de la mère qui apprend très tôt à prendre en charge son bébé dans l'environnement familial [4]. En Tunisie, cette attitude est pratiquée par certaines équipes mais aucune évaluation n'a été réalisée jusqu'à ce jour. L'objectif de notre étude était d'évaluer la faisabilité de la courte hospitalisation dans notre pays et d'identifier les facteurs de risque de ré hospitalisation.

## Méthodes

Il s'agit d'une étude prospective menée au service C du Centre de Maternité et de Néonatologie de Tunis (CMNT), centre universitaire de niveau 3, sur une période de 6 mois, allant de Septembre 2011 à Août 2012, incluant toutes les patientes sorties de l'hôpital à J1 du post-partum. Cette étude a été approuvée par le comité du CMNT.

### Critères d'inclusion

Ont été incluses dans l'étude: les patientes désirant faire partie de l'étude et ayant exprimé leur consentement éclairé par une décharge signée; les patientes habitant dans un rayon de moins de 30km de l'hôpital; les patientes ayant les moyens de revenir à tout moment en cas de besoin; les patientes ayant un examen physique normal à J1 du post-partum (température et tension artérielle normales, pouls normal, examen des seins normal, pas d'hématome du périnée ou de la cicatrice de césarienne, un bon globe, pas de saignement anormal); les patientes pouvant prendre leur traitement à domicile (anticoagulation prophylactique); les patientes bénéficiant du soutien familial (notamment la présence de la mère ou de la belle mère pendant la première semaine); les patientes joignables par téléphone; l'âge, la parité, la voie d'accouchement ainsi que le rétablissement du transit après un accouchement par césarienne n'ont pas été pris en compte dans les critères d'inclusion.

### Critères d'exclusion

Ont été exclues de l'étude: les patientes n'ayant pas compris les signes qui doivent les inciter à consulter à nouveau; les mères célibataires; les grossesses et les accouchements compliqués de pré-éclampsie sévère, d'hémorragie grave de la délivrance traitée par sulprostone ou chirurgicalement, les césariennes jugées difficiles par l'opérateur ou compliquées de lésion urologique ou digestive et les accouchements compliqués de déchirures périnéales du 3^ème^ ou du 4^ème^ degré. Pour chaque patiente incluse dans l'étude on a préconisé une auto-surveillance à domicile par une prise quotidienne de la température. Un régime liquide a été conseillé jusqu'à rétablissement du transit pour les patientes qui ont accouché par césarienne. Les patientes devaient consulter à nouveau en cas de fièvre, de douleurs pelviennes ou d'absence de rétablissement du transit dans les 48h suivant l'accouchement.

Une consultation systématique au dixième jour du post-partum a été préconisée chez toutes les patientes, qu'elles aient accouchées par voie basse ou par césarienne. Durant cette consultation, l'interrogatoire a évalué le degré de satisfaction de la mère par la courte hospitalisation ainsi que son état psychologique en essayant de dépister des signes de dépression du post-partum. On a précisé le déroulement de l'allaitement et les causes d'un éventuel sevrage précoce. Un examen du périnée a été réalisé chez les femmes ayant accouché par voie basse et un examen de la cicatrice opératoire pour les femmes ayant accouché par césarienne, ainsi qu'un examen systématique des seins. Toute patiente qui consultait aux urgences avant le dixième jour, quelle que soit sa plainte, devait être systématiquement ré hospitalisée. Nous avons calculé des fréquences simples et des pourcentages pour les variables qualitatives. Nous avons calculé des moyennes, des médianes et des écarts-types et déterminé les valeurs extrêmes pour les variables quantitatives. Les comparaisons des moyennes ont été effectuées au moyen du test t de Student. Les comparaisons des pourcentages ont été effectuées par le test de Chi-deux de Pearson, et en cas de non validité de ce test, la comparaison s'est faite par le test exact de Fisher. Les données recueillies ont été analysées au moyen du logiciel SPSS version 11.5. Le seuil de signification a été fixé à 0,05.

## Résultats

L'âge moyen de nos patientes était de 28.4 ± 5.2 [extrêmes: 16 - 42 ans]. La majorité des patientes étaient âgées entre 25 et 29 ans (534 patientes soit 44.3%). La gestité moyenne était de 2 ±1,4 [extrêmes: 1 - 10]. La parité moyenne de nos patientes était de 1.7 ± 0.9 [extrêmes: 1 - 7]. Le taux des primipares était de 56.4% et le taux de grandes multipares (parité ≥5) était de 1.5%. Un tiers des patientes (32.4%) avaient un utérus cicatriciel avec un minimum de 1 césarienne (13.9% de nos patientes) et un maximum de 5 (0.5% des patientes). La grossesse était compliquée de diabète dans 9% des cas, de prééclampsie dans 7.5% des cas et d'une association diabète - prééclampsie dans 5.5% des cas. D'autres pathologies ont été notées telles qu'une anémie (4%), une thrombopénie (1%), un retard de croissance intra-utérin (1%) et un placenta preavia (0.5%). Les données relatives à l'accouchement sont résumées dans le Tableau 1. Le terme moyen de l'accouchement était de 38.5±1.6 semaines d'aménorrhée (SA) [extrêmes: 33 - 42 SA]. L'accouchement était par césarienne dans 42% des cas et par voie basse dans 58% des cas. Pour les patientes qui ont accouché par césarienne, 44% étaient sorties avant le rétablissement du transit.

**Tableau 1:** Données relatives à l'accouchement

Terme moyen de l’accouchement (SA)	38.5 ± 1.6
Taux de déclenchement artificiel du travail	25.8 %
Taux de Césariennes	42 %
Taux d’accouchements instrumentaux	3.98
Taux de Déchirures périnéales	2.6
Poids moyen à la naissance (g)	3328 ± 523
Taux d’admissions en néonatologie	3.48%

**Examen au 10éme jour du post-partum**: Quarante-huit patientes (3.98%) ne se sont pas présentées à la consultation du 10^ème^ jour du post-partum. Toutes les patientes ont été contactées par téléphone et n'ont rapporté aucune plainte. Dans 0.8% des cas (10 patientes) on a objectivé un retard simple de cicatrisation de la plaie de césarienne sans infection. L'épisiotomie était bien cicatrisée chez toutes les patientes. L'examen des seins était normal dans 100% des cas. Le taux de patientes allaitantes à J10 était de 99.5% (1200 patientes).

**Ré hospitalisation**: Le taux de ré hospitalisation était de 0.99% (12 patientes). Parmi ces patientes, 2 avaient accouché par voie basse et 10 par césarienne. Huit patientes (66.7%) ont consulté dans les 48 heures après la sortie et 4 patientes (33.3%) ont consulté 72 heures après la sortie. La durée moyenne de la ré hospitalisation était de 26h [extrêmes: 14 - 72 h]. Le principal motif de consultation était le ballonnement abdominal secondaire à l'iléus paralytique postopératoire (50% des cas). Ces patientes ont été ré hospitalisées pendant un maximum de 24 heures, l'examen physique ainsi que les examens complémentaires n'ont trouvé aucune anomalie; 3 ont eu une aspiration naso-gastrique devant une gêne importante, les autres ont rétabli spontanément leur transit. Les autres causes de ré hospitalisation étaient: une embolie pulmonaire (1 patiente): la patiente a consulté pour une dyspnée apparue 72 heures après la sortie. Le diagnostic a été confirmé par angioscanner thoracique et la patiente a été traitée par héparine à bas poids moléculaire à doses curatives relayée par l'anti-vitamine K. C'est une deuxième pare sans antécédent pathologique qui a accouché par césarienne en urgence pour stagnation de la dilatation et qui avait pourtant pris correctement son anti coagulation prophylactique; des céphalées post rachianesthésie en rapport avec une brèche duremérienne traitées par hydratation, caféine et blood patch (1 patiente); un hématome périnéal (1 patiente). La patiente a reconsulté pour des douleurs au niveau de l'épisiotomie 24 heures après sa sortie; l'examen clinique et l'échographie ont mis en évidence un hématome de 6cm. Elle a eu un traitement conservateur par embolisation radiologique avec une bonne évolution; une fièvre (3 patientes): chez la première patiente la fièvre s'est déclarée au 4^ème^ jour post-partum. Le diagnostic retenu était une endométrite. Chez la 2^ème^ patiente on a retenu comme diagnostic étiologique une infection de la plaie opératoire de césarienne. Enfin, chez la 3^ème^patiente, on a retenu comme diagnostic étiologique une infection urinaire. Les 3 ont bien évolué après traitement adapté.

**Facteurs de risque de ré hospitalisation**: Les facteurs de risque de ré hospitalisation, statistiquement significatifs, identifiés dans notre étude étaient: la césarienne (p=0,004), la césarienne en urgence (p=0,016), l'anémie (p<0,001) et la thrombopénie (p=0,003) (Tableau 2).

**Tableau 2:** Récapitulatif des facteurs étudiés

	Groupe RH+	Groupe RH-	P
Age moyen	27,6	28,4	0.611
Gestité moyenne	2,17	2,01	0.714
Parité moyenne	1,67	1,73	0.814
Terme d’accouchement moyen	38,6	38,5	0.771
Taux d’accouchements par césarienne	83,3%	16,7%	0.004
Taux d’épisiotomie	100%	97,4%	0.82
Taux de déchirures périnéales	0%	2,6%	0.82
Taux de césariennes en urgence	100%	61,4%	0. 016
Taux initial d’hémoglobine	9,2 g /dl	11,6g /dl	<0.001
Taux initial de plaquettes	227465	298500	0.003 S

**Groupe RH+ :** groupe des patientes ré hospitalisées après une sortie précoce

**Groupe RH- :** groupe de patientes non ré hospitalisées après une sortie précoce

## Discussion

### Définition de la courte hospitalisation

La courte hospitalisation du post-partum ou sortie précoce n'a pas de définition univoque dans la littérature. Sa durée varie de 24 à 72 heures selon les auteurs [3, 7–10]. Dans notre étude nous avons retenu la sortie à J1 du post-partum comme définition de la sortie précoce. La durée standard d'hospitalisation en post-partum en Tunisie étant de 24 à 48 heures pour les accouchements par voie basse et de 48 à 96 heures pour les accouchements par césarienne.

### Caractéristiques de la population et de l'accouchement

Dans notre étude nous avons un taux élevé de femme ayant accouché par césarienne. Ceci peut être expliqué par le fait qu'une grande partie des femmes qui accouchent dans notre centre sont référées pour des indications de césariennes et que notre centre est le centre universitaire de niveau III de référence en Tunisie et draine de ce fait les grossesses à risque d'une grande partie du pays. Dans notre étude le taux de primipares était de 56.4%. Dans la littérature trois études uniquement rapportent un taux de primipares supérieur à 50% [11–13]. En effet certains auteurs sont réticents vis-à-vis de la sorti précoce des primipares vus leur manque d'expérience. Dans notre étude nous avons exclu les mères célibataires et les patientes qui n'avaient pas de soutien familial (présence de la mère ou de la belle-mère). En effet dans notre société les parturientes ont souvent une personne de la famille qui les assiste pendant au moins les premiers jours suivant l'accouchement. Nous avons exigé cette présence dans notre étude vu qu'il n'existe pas de visites de sage-femme à domicile en Tunisie.

### Les réadmissions maternelles

Le taux de réadmissions maternelles n'est pas augmenté en cas de sortie précoce, lorsqu'on compare les études ayant exclu les femmes à haut risque et celles qui les ont incluses [3, 7, 9, 10, 14, 15]. Ce taux varie dans la littérature entre 1.8% et 3.5% [1, 3, 9, 11, 14]. Il était de 0.99% dans notre étude. La principales causes de réadmission dans la littérature est la fièvre (plus de 50% des cas), en rapport le plus souvent avec une endométrite ou une mastite; rarement une bartholinite [9, 14, 16]. Les problèmes au niveau de l'épisiotomie, (essentiellement les hématomes, les lâchages de points et les douleurs), constituent un autre motif de consultation fréquent et justifient près de 25% des admissions [9, 14, 16]. Aucune complication grave mettant en jeu le pronostic vital n'a été rapportée dans la littérature, seul Boulvain et al. ont rapporté un décès maternel inexpliqué dans les 6 mois suivant la sortie [9]. Il n'a pas été rapporté dans les différentes séries de différence statistiquement significative en termes de consultation aux urgences entre les patientes ayant bénéficié d'une courte hospitalisation et celles ayant eu une hospitalisation standard [10, 16–18].

### Evaluation psychologique de la mère

Il y a eu beaucoup de controverses au le sujet de la courte hospitalisation et ses risques psychologiques potentiels pour la mère [19]. Dans les différentes études, le taux de fatigue et d'insomnie était comparables entre les groupes courte hospitalisation et hospitalisation standard [9, 11, 13, 20]. Toutefois, l'effet négatif le plus redouté serait une augmentation du taux de dépression du post-partum [1, 5]. Plusieurs auteurs ont réalisé une évaluation psychologique de la mère pour dépister cette complication qui constitue rarement un motif de consultation et qui risque donc de passer inaperçue. Ces auteurs ont dépisté la dépression maternelle du post-partum, en utilisant différents scores [9–11, 14, 17, 21]. Les résultats de ces études sont toutefois discordants [9–11, 14, 17]. Dans notre série nous n'avons relevé aucun cas de dépression du post-partum. Aucune patiente n'était d'humeur dépressive. Néanmoins nous n'avons pas utilisé de score d'évaluation précis ce qui aurait pu nous faire méconnaître des dépressions légères.

### Influence sur l'allaitement

La plupart des études qui se sont intéressées au sujet n'ont pas trouvé une corrélation entre la durée de séjour en post-partum et le taux d'arrêt de l'allaitement maternel [8, 22–24]. Dans notre étude il n'y avait pas de groupe contrôle pour pouvoir évaluer l'influence de la sortie précoce sur la poursuite de l'allaitement. Nous avons juste évalué l'allaitement à J10 du post-partum. A cette date, 99.5% des femmes étaient encore allaitantes. Le but de cette évaluation précoce était essentiellement de dépister des problèmes rencontrés ou des difficultés pouvant amener les femmes à arrêter l'allaitement.

### Les admissions néonatales

La plupart des études publiées ont exclu les femmes dont le nouveau-né était admis en pédiatrie [25]. Nous n'avons pas considéré, dans notre étude, les admissions néonatales comme critère d'exclusion. La sortie du nouveau-né étant indépendante de celle de la mère. Dans notre série, 42 patientes sont sorties sans leurs bébés. Parmi ces femmes, 3 uniquement n'ont pas allaité leurs bébés: l'une avait un nouveau-né prématuré avec un faible poids de naissance et les deux autres avaient des bébés porteurs d'une hernie diaphragmatique décédés dans les 48 heures suivant l'accouchement. Pour les autres, elles avaient droit à des visites quotidiennes et pouvaient soit allaiter le bébé soit fournir leur par un tire-lait. La durée moyenne de l'hospitalisation néonatale était de 2,8 jours [extrêmes: 1 - 6 jours]. Nous n'avons pas évalué le taux de réadmissions néonatales des bébés sortis précocement avec leur maman. Néanmoins dans une méta-analyse incluant 10 études publiées en 2002, Brown et al. n'ont pas retrouvé d'augmentation du taux de réadmissions néonatales après une sortie précoce durant les 3 à 8 semaines suivant l'accouchement [4].

### Intérêt économique

L'intérêt économique sur le coût global de la courte hospitalisation a été bien démontré dans la littérature [11, 26–29]. Même si les résultats des études sur le coût après la sortie sont discordants; le coût global de l'accouchement comprenant les dépenses de l'hospitalisation et la prise en charge après la sortie reste toujours moindre en cas de courte hospitalisation [11, 26]. Dans notre étude, nous avons estimé les gains en fonction du prix moyen d'une journée d'hospitalisation. Si nous considérons le standard tunisien qui est de 24 à 48 heures d'hospitalisation pour les accouchements par voie basse et de 48 à 96 heures pour les accouchements par césarienne, les gains estimés seraient de 140 à 240 € par patiente. La prise en charge après la sortie ne génère pas dépenses supplémentaires en cas de courte hospitalisation puisque l'état ne propose pas de sage-femme à domicile. Ainsi, nous pouvons estimer le gain pour notre service (qui gère environ 3000 accouchements/an) à 420 000 à 840 000 € par an.

### Les patientes perdues de vues

Le taux des patientes perdues de vu varie dans la littérature entre 0 et 12% [7–10, 14, 17, 20, 30] et il est le plus souvent inférieur à 4% [8–10, 14, 20, 30]. Dans notre série 3.98% des patientes ne sont pas revenues à la consultation du 10^ème^ jour. Toutes ces patientes ont été contactées par téléphone et ont affirmé qu'elles allaient bien et qu'elles avaient consulté au dispensaire le plus proche pour l'ablation des points. Ceci prouve que ces patientes n'étaient pas assez sensibilisées à l'intérêt de cette consultation systématique du dixième jour et montre aussi l'intérêt de la notion de proximité comme critère d'inclusion dans un protocole de sortie précoce [31].

## Conclusion

La courte hospitalisation du post-partum est une option qui permet de réduire significativement les dépenses d'hospitalisation tout en étant sure pour la maman et le nouveau-né, sous réserve du respect des critères de sélection. Dans notre étude, les facteurs de risque de réadmission maternelle étaient: la césarienne, la césarienne en urgence, l'anémie et la thrombopénie.

### Etat des connaissances actuelles sur le sujet

- La sortie précoce en post-partum est une pratique de plus en plus courante dans le monde;
- Il s'agit d'une pratique sure dans les pays développés grâce aux soins et aide offerts par les sages-femmes à domicile.

### Contribution de notre étude à la connaissance

- La sortie précoce en post-partum semble une pratique sure dans les pays où existe encore une forte cohésion familiale;
- La sortie précoce en post-partum apporte un gain économique évident dans les pays qui n'offrent pas de sages femmes à domicile et qui présentent toutefois une structure sociale caractérisée par une forte cohésion familiale.
